# Comparison of Definitional skills in school-age children with cochlear implants and normal hearing peers

**DOI:** 10.22037/ijcn.v15i1.22175

**Published:** 2021

**Authors:** Shima HOSSEINABADI, Talieh ZARIFIAN, Robab TEYMOURI, Enayatollah BAKHSHI

**Affiliations:** 1 Department of Speech Therapy, University of Social Welfare and Rehabilitation Sciences, Tehran,Iran.; 2 Pediatric Neurorehabilitation Research Center, University of Social Welfare and Rehabilitation Sciences, Tehran, Iran; 3 Department of Biostatics, University of Social Welfare and Rehabilitation Sciences, Tehran, Iran.

**Keywords:** Language Development, Vocabulary, Cochlear Implant, Metalinguistics, Children

## Abstract

**Objective:**

The auditory experience is important because makes a major contribution to the development of speech, language, cognitive, and social skills. Knowledge of the lexicon has been increased throughout life. Input factors and linguistic and metalinguistic knowledge are effective factors in the acquisition of definitional skills. This study was done to investigate definitional skills in cochlear-implanted (CI) children and their typically developing (TD) peers.

**Materials & methods:**

A total of 46 third-grade primary school children (16 with cochlear implants and 30 their TD peers) were recruited. The verbal definitional task included 14 common high-frequency nouns and 11 common high-frequency verbs. All definitions were scored for both content (semantic) and grammatical forms. Statistical analysis was conducted to compare the definitional skills between the two groups.

**Results:**

There were significant differences between CI children and their TD peers for word definition skills in both categories of content and form (p<0.001). The results showed the mean scores of content and

form aspects of word definition in the TD group were approximately twice higher than the CI ones (M±SD=133±28 and M±SD= 78±23, respectively).

**Conclusion:**

Children with CI may have trouble with definitional skills. It seems that the lower scores of CI children in definitional skills were due to a lack of auditory experience. Considering interventions on definitional skills in CI children is suggested.

## Introduction

Hearing impairment (HI) is the most common congenital defect. Of the 360 million people thought to have hearing impairment in 2011, approximately 32 million were children younger than 15 years of whom 7.5 million were younger than 5 years (1) (2). In general, when devastating HI develops at a young age, it impedes normal speech and language development (3).

Cochlear implantation is one of the most useful ways to empower those who are hearing-impaired and offers them the most advanced technical solution for the enhancement of deep sensorineural hearing loss. Children who use cochlear implants (CI) have already lost a vital part of language acquisition prior to their implantation, and language acquisition has naturally become problematic for these children at all levels (4). Accordingly, on average, the depth of the vocabulary knowledge of hearing-impaired children is lower than of their typically developing (TD) counterparts (5).

The individual’s vocabulary store grows and develops constantly throughout life and word-defining is also part of the skills that depend on vocabulary learning and size (6). Every child should learn to deﬁne vocabulary for academic achievement as well as for practical use in daily conversation to avoid miscommunication (7).

Studies on word-defining skills consider the two aspects called ‘content’ and ‘form’ in the development of vocabulary skills and each aspect can have a different developmental path (7). The development of word-defining skills is a gradual process that begins when the person enters school and continues into adulthood (8). One method to evaluate vocabulary to assess the depth of children’s knowledge about the target term is asking children to define the word or tell whatever he knows about the meaning of the target word (9).

Researchers who investigate language as a product of the human mind, use observable behaviors in the relevant tasks to deduce mental functions due to the lack of direct access to inner-mental activities. Word-defining is one of the tasks that contribute to the organization of meanings in mental vocabulary. Word-defining skills are associated with linguistic and cognitive skills (10). In this task, the individual has to define a given word. This task measures the contestant's metalinguistic abilities (8). In word-defining, both nouns and verbs are defined. It is generally understood that word-defining progresses from functional responses in childhood toward more conceptual ones in adulthood.

We know that deﬁnitional skill slowly improves in both content and form (syntactic structure) during the school education and adolescent years. Content and form definition may follow different developmental routes (11). In terms of content, it means the expression of the semantic features of a word that distinguishes it from other related words in that category (12), and in terms of structure, it involves the formulation of a definition to precisely and fully conveys mental information about that word (12). 

The organization of meanings in mental vocabulary is effective in predicting children's success in their participation in society, such as schools (8). Children acquire basic language skills before reaching school age and can use these skills to communicate with others in different social settings and easily master different degrees of linguistic complexity. Yet, for unknown or known reasons (including sensory problems, such as auditory impairment), the process of learning language and its components, including vocabulary development, is delayed or disrupted in some children (12). The auditory sense is an important sense that makes a major contribution to the development of speech, language, and cognitive skills (13).

Some professionals, particularly general practitioners, sometimes advise parents of preschool children that they will grow out of a language problem after CI procedure, and no intervention is required; however, some clinical practitioners believe that intervention should be offered as early as possible because it is more cost-effective to shape a developing system (14). They assert that despite progress in semantic, syntactic, and morphological areas, hearing-impaired children still appear poor in the proper use of language and require special training. When this training is provided late, these children will face greater communication problems and require further and longer training (15). However, little is known about the effect of hearing condition on vocabulary development and also it has not yet been known what certainly causes the differences in vocabulary knowledge competence among hearing loss children. Knowledge about the process of vocabulary development and the factors that can affect vocabulary progress can help to promote academic achievement (16). Children with limited and insufficient language skills not only show deficits in active verbal memory and language learning and processing but are also unable to develop and broaden their vocabulary and understand and express semantic differences in words with close meanings (17). It is therefore important to determine the performance of children with HI in word-defining skills. Many studies have been conducted on word-defining skills in children of different age groups from different countries; however, less attention has been paid to word-defining skills in children with HI.

Marinely & Janson investigated word-defining skills in children with specific language impairment (SLI). Fifteen children with SLI and 15 control children were asked to define ten high-frequency words, and their definitions were assessed in terms of content and structure. The SLI group scored significantly lower in both content and structure compared with the control group precisely because of their disrupted access to words or deficiency in metalinguistic knowledge (8).

In another study, Marinely & Jonson investigated word-defining for verbs and nouns in 30 primary school children. The target words included ten high-frequency nouns, such as ‘apple’ and ‘child’, and ten high-frequency verbs, such as ‘climb’ and ‘eat’. The definitions were assessed by content (semantic) and structure (syntax). Their results showed no significant differences in the definitions provided for nouns and verbs in terms of content, but in syntactic terms, the scores obtained for the noun definitions were significantly higher than the verb definitions (7).

In a study conducted by Gavriilidou, word-defining skill development was investigated in Greek 38 pre-school children aged 4.2 to 6.5 years old. The word-defining task consisted of 16 objective and abstract nouns, verbs, and adjectives. The results revealed that the responses progressed from objective and functional to more formal and objective with age. Another objective of the study was investigating the effect of gender on word-defining skills, but the gender variable appeared to have no significant effects on this skill (18). 

Word-defining abilities as skills to reflect important data on learning and feedback on the meaning of words and the organization of concepts in the vocabulary store can help compare children with HI and their normal peers in terms of semantic development and the organization of concepts. Considering that word-defining is one of the skills, in which children at the age of learning a language and beyond are involved, investigating this skill in terms of structure and content in children with HI compared with normal children seems necessary. Thus, by describing the nature of word-defining in this group of children, appropriate empowerment strategies can be adopted, and in the case of delays, compensatory measures can be developed for children with HI. 

The present study was done to study this skill in terms of content and structure in children using CIs by word-defining tasks (nouns and verbs) and compare the results with their normal peers. 

## Materials & Methods

**Participants**

In the present study, the participants in the CI group were selected through convenience sampling from those referring to cochlear implantation centers in Tehran, Iran. Based on previous studies and the relevant equation, the sample size was estimated at 16 for the children with CI group and 30 for the TD group. The CI group included third-year primary school children using CI (eight girls and eight boys) selected according to the inclusion criteria, i.e. unilateral CI and hearing aid on the other ear, attending conventional schools, attending 100 CI training sessions, no mental problems, a passing score in the pre-school exam, and the lack of fail scores in the previous years. All children with CI used auditory-oral communication modes and they have CI surgery before the age of 3 years.

Thirty TD children (15 boys and 15 girls) were selected randomly from third-year primary-school children according to the inclusion criteria, i.e. no hearing, speech, or language problems within the normal limits and the lack of fail scores in the previous years. 

Written consent was obtained from the families prior to the test. This research was approved by the ethics committee of the University of Social Welfare and Rehabilitation Sciences. 

Noun definition task

The noun definition task developed by Mohammadi was used in the noun-defining section, with scoring based on the study by Marinely & Jonson (8). This test measures students’ word-defining skills in seven areas, including animals, jobs, fruits, places, body parts, eating tools, and transportation vehicles. Each of these seven areas contains two words that are normally used by children at the primary school level. These 14 words are objective and frequently used nouns that only bring one meaning to mind and can be defined in various ways –from simple to complex (12) (Appendix A).

The validity of the noun-defining task in terms of content and structure was determined by calculating the inter-rater correlation. The validity of the content part was 71% in the first trial and 81% in the second, and the validity of the structure part was 80% in the first trial and 91% in the second (12). 

Verb definition task

The validity of the verb definition task was assessed using a researcher-made test. Eleven frequently used verbs in Persian were selected and the content validity of the verbs and the scoring of the test were confirmed by ten speech therapists (CVR=0.89). The verb-defining task consisted of two parts, i.e. content and structure. The inter-rater agreement was measured using Kendal's method (r=1) (Appendix B).

## Results

Given the scoring table described, the content and structure scores of noun-defining and verb-defining were both calculated for each child, and the total noun-defining and verb-defining scores (in terms of content and structure) were then measured, as well. The mean and standard deviation for each academic year were then calculated.

Following the Shapiro-Wilk test and given the non-normal distribution of the data, Mann–Whitney U test was used for the pair-wise comparisons.

Comparing the CI third-year primary-school children and their TD peers in terms of the content of noun-defining showed a significant intergroup difference (P<0.001) (Table 1). 

**Table 1:** Comparing the scores of form and content aspects of the noun

**Participants**		**Content**				**Form**			
**Group**	**Number**	**Mean** **(SD)**	**Lower limit**	**Upper limit**	**P-value**	**Mean** **(SD)**	**Lower limit**	**Upper limit**	**P-value**
**TD **	**30**	**46.03** **(13.00)**	**41**	**50**	**0.001**	**46.00** **(9.00)**	**43**	**50**	**0.001**
**CI**	**16**	**23.00** **(10.03)**	**18**	**29**		**26.06** **(8.00)**	**21**	**30**	

TD: Typically Developing, CI: Cochlear Implant, SD: Standard deviation

The mean score in the TD group was almost double the CI group. The highest and lowest scores were 63 and 12 in the TD group and 47 and 11 in the CI group.

Comparing the CI children and their TD peers in terms of the structure of noun-defining also showed a significant intergroup difference (P<0.001) (Table 1). 

Also, comparing the CI children and their TD in terms of the content and form of verb-defining showed a significant inter-group difference (P<0.001) (Table 2).

**Table 2:** Comparing the scores of form and content aspects of the verb

**Participants**		**Content**				**Form**			
**Group**	**Number**	**Mean** **(SD)**	**Lower limit**	**Upper limit**	**P-value**	**Mean** **(SD)**	**Lower limit**	**Upper limit**	**P-value**
**TD**	**30**	**18.06** **(6.00)**	**15**	**20**	**0.001**	**22.00(4.09)**	**20**	**23**	**0.001**
**CI**	**16**	**10.00** **(2.00)**	**8**	**11**		**18.00(5.00)**	**15**	**21**	

TD: Typically Developing, CI: Cochlear Implant, SD: Standard deviation

The data obtained from comparing the CI children and their TD peers in terms of the structural aspect of verb-defining also showed a significant intergroup difference (P<0.001) (Table 2).

Table 2 shows that there was a greater difference between the two groups in the mean total (in terms of content and structure) scores obtained for nouns compared with verbs, but both groups generally scored higher in terms of noun-defining compared with verb-defining.

Table 3 shows a significant difference between the TD and CI groups in the total score of noun-defining (P<0.001).

**Table 3:** The total score of the content and form of verb and noun

**Participants**		**Noun**				**Verb**			
**Group **	**Number**	**Mean ** **(SD)**	**Lower limit **	**Upper limit**	**P-value**	**Mean ** **(SD)**	**Lower limit**	**Upper limit**	**P-value**
**TD **	**30**	**92.00(22.00)**	**84**	**101**	**0.001**	**40.00(9.00)**	**36**	**43**	**0.001**
**CI**	**16**	**49.00(17.00)**	**40**	**59**		**28.00(6.00)**	**24**	**32**	

TD: Typically Developing, CI: Cochlear Implant, SD: Standard deviation

Table 3 also represents a significant difference between the TD and CI groups in the total score of verb-defining (P<0.001).

As shown, noun-defining showed higher scores compared with verb-defining in the TD group, and this pattern was also observed in the CI group. Although the noun- and verb-defining scores were lower in the CI group than the TD group, word-defining skills appear to follow the same pattern in CI children as in TD children.

Table 4 presents a significant difference between the TD and CI groups in terms of total scores (P<0.001).

**Table 4:** The total score of verb and noun

Group	Number	Mean (SD)	Lower limit	Upper limit	P-value
TD	30	133.00(28.00)	122.00	143.00	0.001
CI	16	78.00(23.00)	66.03	90.00	

TD: Typically Developing, CI: cochlear Implant, SD: Standard deviation

## Discussion

Word-defining is a verbal skill, in which the individuals describe a word using other words (19). The general purpose of the present article was to investigate and compare word-defining skills in third-year primary-school children using CI and their TD peers to determine the features of word-defining skills in CI children and use the results to prepare a treatment program for improving linguistic skills in children with HI. 

According to the results, the significant mean values obtained by the comparison of the content and structural aspects of nouns and verbs revealed differences between the CI and TD groups in different aspects of language, as the TD group was more advanced than the CI group in its progress in different aspects of the language. 

A review of previous studies suggests deficiencies in word-defining skills in children with language impairment (8) and (12) their healthy peers. CI children perform poorer in language development and phonological knowledge compared with their healthy peers (20). The results of the content aspect of noun- and verb-defining showed that the mean scores in the TD group were twice higher than in the CI group, which can be due to the higher semantic representation in the TD group, which led to fuller responses. The review of the structural aspect of nouns and verbs showed higher mean scores in the TD group compared with the CI group, and these differences suggest that the TD group had greater mastery over the proper ways of answering and used fuller linguistic structures to convey the meanings of the nouns and verbs. These differences are indicative of the linguistic superiority of the TD group over the CI ones. The development of word deﬁnitions is severely facilitated by the situations of hearing, models of education, and manner of practicing. Probably children are more exposed to the models of nouns than verbs at schools (21).

In line with the results obtained by Marinely & Jonson, the mean scores of the content and structural aspects of noun-defining were higher than the mean scores of verb-defining in both groups, and the children had better definitions structurally for nouns compared with verbs. Providing the proper syntactic form for nouns is easier than verbs for children, and they learn helpful practices at school; nouns may frequently lead to the activation of a certain categorical or superordinate term. This categorical term can have an organizing elements’ role, by which a deﬁnition can be formed (11).

The present findings also revealed that word-defining was different for nouns compared with verbs. Noun deﬁnitions are less variable in form than a verb and adjective deﬁnitions, but verbs can have a developed (i.e. conventional) deﬁnitional form like nouns (22). 

It is easier for children to express word content than using conventional form definitions. When a deﬁnition has a syntactic form of the word being deﬁned, the conventional form is present (11). Nouns that contained structured classification are stored in totally interconnected networks. The hierarchical nature and interconnected organization of the nouns may result in easier, more transparent semantic relations than verbs and adjectives. Lexical organization for verbs and adjectives may be less transparent and predictable. Verbs and adjectives may be displayed in a nonhierarchical manner (23). Subcategories of the terms for verbs are less clear than nouns. For example, it is clear that the superordinate for a dog is animal, but superordinate for the verbs, like play and eat are less clear. Verbs may be organized in different ways than nouns, each verb is considered to be a noun-dependent lexical category: A verb is linked to other verbs and nouns. The speed of learning verbs in children is slower than a noun and it shows that verbs may be more difﬁcult to learn than nouns (24).

The children's answers in this study were assessed in terms of content and structure to obtain a more comprehensive view of the development of this skill in CI and TD children. CI children showed significant differences compared with the TD group in terms of noun-defining, which could partly be due to their limited vocabulary than their peers. 

According to the presented findings, language difficulties in CI children do not allow the formation and presentation of a complete process of word-defining. 

This study, especially regarding semantic and structural aspects, not only strengthens the definition of familiar concepts but may also help with the development of the definition of new concepts in CI children and resulting in academic and communicational achievements in this group of children.

Studies are often faced with uncontrollable limitations and variables, and the present study was no exception to this rule. One of the limitations of this study was the small sample size in the CI group, as a result of which some hearing variables, such as age at cochlear implantation and matching according to the type of prosthesis used was not possible. This issue is recommended to be considered in future studies.

**Appendix A:** Items of the noun definition task

**Fruits**	**Animals**	**Places**	**Job**	**Vehicles**	**Meals**	**Organs of the body**
Apple	Horse	School	Teacher	Airplane	Spoon	Leg
Pomegranate	Crow	Mosque	Doctor	Train	Glass	Hand

**Appendix B:** Items of the verb definition task

Break	Write	Play	Wash	Sleep	Buy	Run	Comb	Drop	Paint	Wear

## Conclusion

Children with CI showed lower performance in definitional skills. It seems that the potential cause for lower scores of CI children in definitional skills was the lack of auditory experience. Considering interventions on definitional skills in CI children is suggested.
